# Supplementary material for: Understanding the impact of the SARS-COV-2 pandemic on hospitalized patients with substance use disorder
Source: PLoS One. 2021 Feb 26;16(2):e0247951. doi: 10.1371/journal.pone.0247951 (PMC7909702; doi:10.1371/journal.pone.0247951)
Supplement: S1 File — Survey for participants with substance use disorder, hospitalized during the SARS-COV-2 pandemic. (PDF) [file pone.0247951.s001.pdf]

COVID SUD Survey

Record ID

____

Referral Source

- ☐ EPIC
- ☐ Person
- ☐ Both

Participant not participating because:

- ☐ Participant refused
- ☐ Not able to participate in interview due to cognitive deficit
- ☐ Non-English speaker
- ☐ Patient currently incarcerated
- ☐ Patient has deceased
- ☐ Unable to participate due to medical condition
- ☐ Judgement of research staff

Why did the participant refuse?

____

Exclusion date

____

Survey start time

____

Survey end time

____

Is there one place in particular, like a doctor's office or clinic, where you usually go to when you need medical care?

- ☐ Yes
- ☐ No
- ☐ Don't know

Next is a set of questions about your alcohol and drug use, etc. Before we start, I want you to know that we are asking these questions to better understand how COVID-19 may impact you; there is no judgement here about substance use. The questions generally ask about the past 30 days.

In the past 30 days, how many days did you drink ANY alcohol?

____

In the past 30 days, how many days did you have at least 5 drinks (if you are a man) or at least 4 drinks (if you are a woman)? [One drink is considered one shot of hard liquor (1.5 oz) or 12-ounce can/bottle of beer or 5-ounce glass of wine.]

____

In the past 30 days, how many days did you use any of the following drugs:

Marijuana (cannabis, pot, weed)	__________________
Sedatives and/or Tranquilizers (benzos, Valium, Xanax, Ativan, Ambien, barbs, Phenobarbital, downers, etc)?	__________________
Cocaine and/or Crack?	__________________
Methamphetamine?	__________________
Other Stimulants (other amphetamine, Dexedrine, Ritalin, Adderall)?	__________________
Opiates (Heroin, morphine, Dilaudid, Demerol, Oxycontin, oxy, codeine (Tylenol 2,3,4), Percocet, Vicodin, Fentanyl, etc?)	__________________
Inhalants (glues, adhesives, nail polish remover, paint thinner, etc)?	__________________
Other drugs (steroids, non-prescription sleep and diet pills, Benadryl, Ephedra, other over-the-counter or unknown medications)?	__________________
In the past 30 days, how much were you bothered by cravings or urges to drink alcohol or use drugs?	◯ Not at all<br>◯ Slightly<br>◯ Moderately<br>◯ Considerably<br>◯ Extremely
Please describe how you feel about your alcohol use right now:	◯ I do not want to think about cutting back or quitting<br>◯ I want to cut back but I am not ready to quit<br>◯ I want to quit<br>◯ I quit before I was admitted to the hospital
Please describe how you feel about your amphetamine or methamphetamine use right now:	◯ I do not want to think about cutting back or quitting<br>◯ I want to cut back but I am not ready to quit<br>◯ I want to quit<br>◯ I quit before I was admitted to the hospital
Please describe how you feel about your opioid use right now:	◯ I do not want to think about cutting back or quitting<br>◯ I want to cut back but I am not ready to quit<br>◯ I want to quit<br>◯ I quit before I was admitted to the hospital
Please describe how you feel about your other drug use right now:	◯ I do not want to think about cutting back or quitting<br>◯ I want to cut back but I am not ready to quit<br>◯ I want to quit<br>◯ I quit before I was admitted to the hospital

In the past 30 days, how many days did you attend self-help meetings like AA or NA to support your recovery?

____

Does your religion or spirituality help support your recovery?

- ◯ Not at all
- ◯ Slightly
- ◯ Moderately
- ◯ Considerably
- ◯ Extremely

Do you have enough income (from legal sources) to pay for necessities such as housing, transportation, food and clothing for yourself and your dependents?

- ◯ Yes
- ◯ No

What is your zip code where you live?

____

If don't know: What city or town do you live in?

____

Which of the following best describes your current living situation?

- ◯ Own or rent your own apartment or house
- ◯ Staying with a friend or family member
- ◯ Staying in a motel or hotel
- ◯ Staying in a shelter
- ◯ Staying on the streets, a park or in a car
- ◯ Staying in a nursing home or rehabilitation facility
- ◯ Refused

Do you have a cell phone here with you in the hospital? If yes, does it have video chat capability, like FaceTime, Skype, or Zoom?

- ◯ I have a phone with me and it has video chat capability
- ◯ I have a phone with me but it doesn't have video chat capability
- ◯ I have a phone with me but I'm unsure if it has video chat capability
- ◯ I don't have a phone with me
- ◯ Refused

What is the highest grade or year of school you completed?

- ◯ Never attended school or only kindergarten
- ◯ Grades 1 through 8 (elementary)
- ◯ Grades 9 through 11 (some high school)
- ◯ Grade 12 or GED (high school graduate)
- ◯ College 1 to 3 years (some college or technical school)
- ◯ College 4 years or more (college graduate)
- ◯ Refused

What was your gross household income before taxes and deductions, for last year?

- ◯ $0
- ◯ $1-$10,000
- ◯ $10,001 - $20,000
- ◯ $20,001 - $30,000
- ◯ $30,001 - $40,000
- ◯ $40,001 - $50,000
- ◯ >$50,000
- ◯ Don't know
- ◯ Refused

What best describes your marital status? (Let participant answer feely and code the closest choice. If they are having difficulty, offer the choices from the list).	◯ Now married<br>◯ Widowed<br>◯ Divorced<br>◯ Separated<br>◯ Never married<br>◯ Living with a long term partner<br>◯ Refused
What race do you consider yourself to be?	☐ American Indian or Alaskan Native<br>☐ Asian<br>☐ Black or African American<br>☐ Native Hawaiian or Other Pacific Islander<br>☐ White<br>☐ More than one race<br>☐ Unknown<br>☐ Other<br>☐ Refused
Are you of Hispanic origin?	◯ Yes<br>◯ No<br>◯ Unknown
What is your gender?	☐ Male<br>☐ Female<br>☐ Other: Specify<br>☐ Refused
Other: specify gender	__________________________________
Do you consider yourself to be:	☐ Straight (heterosexual)<br>☐ Lesbian<br>☐ Gay<br>☐ Bisexual<br>☐ Asexual<br>☐ Other: specify<br>☐ Refused
Sexual orientation: other specify	__________________________________

We've talked a lot about your drug use and how you feel about it. Now I'd like to ask you some more general questions about your experience of being sick and in the hospital, and how COVID or coronavirus is affecting you. There won't be any choices for answers - please answer freely and as openly as you feel comfortable. We are talking with people in the hospital to learn about your experience in your own words. A few reminders before we begin. First, I am not part of your medical team and anything you share with me is completely confidential. Our research team will not share any information with your medical team unless you ask us to or if you or someone else are at risk of harm. Also, I will be recording this part of the interview; please let me know if you would like me to stop the recording at any time. Lastly, you can stop this interview at any time or decline to answer any question I ask of you. What questions do you have before we begin?

RESEARCH ASSISTANTS: Please feel free to take notes/transcribe as folks speak (in addition to recording), and include any field note observations at end of survey.

Tell me about why you are sick and in the hospital.
Prompt: Tell me about what happened when you started to get sick?

____

Tell me how you've been feeling in the hospital during the COVID crisis

____

3. For many people, COVID has changed their experience of health and healthcare. Can you tell me about your experiences?
Prompt:
a. For some people, addiction providers are calling them in their rooms. If that has happened for you, how has that been?
b. Other hospital/healthcare experience (e.g. masked providers, phone visits)
c. Mental health
d. Family support/Community/Partners?

____

Tell me about how your life before hospitalization was affected by COVID

____

5. For many people, COVID has changed their drug use. Can you tell me how COVID has affected these aspects of your life?
Prompts:
a. Access to drugs and alcohol
b. Settings or ways in which you typically use - for example, using alone, or using different substances (for example, using fentanyl or methamphetamine instead of heroin)
c. Access to harm reductions services like needle exchange

____

6. For some people, COVID has impacted their treatment/or recovery supports. How would you describe your recovery journey?
a. Prompt:
b. How as COVID impacted your substance use treatment?

i. Access to bupe/methadone?
c. How has COVID impacted your ability to access recovery support systems like AA or NA, or peer support?

____

7. For many people, COVID has affected people's lives. How has COVID affected your life?
a. Some people have told me that COVID impacts their:
b. Housing
c. Food services/ free meals
d. Job/ money/ employment

____

8. For some people, COVID has changed their experiences at home, or their relationships with family for friends. How has COVID impacted your relationships with others?
a. Intimate partners
b. Parents or siblings
c. Children/parenting
d. Relationships with friends
e. Community or neighbors

____

What are you biggest concerns as you navigate leaving the hospital during the time of COVID?

____

Is there anything that we haven't talked about today that you think I should know?

____

Field Notes from Researcher

____
