# Supplementary material for: Understanding the impact of the SARS-COV-2 pandemic on hospitalized patients with substance use disorder
Source: PLoS One. 2021 Feb 26;16(2):e0247951. doi: 10.1371/journal.pone.0247951 (PMC7909702; doi:10.1371/journal.pone.0247951)
Supplement: S1 Poster — (PPTX) [file pone.0247951.s002.pptx]

## Slide 1
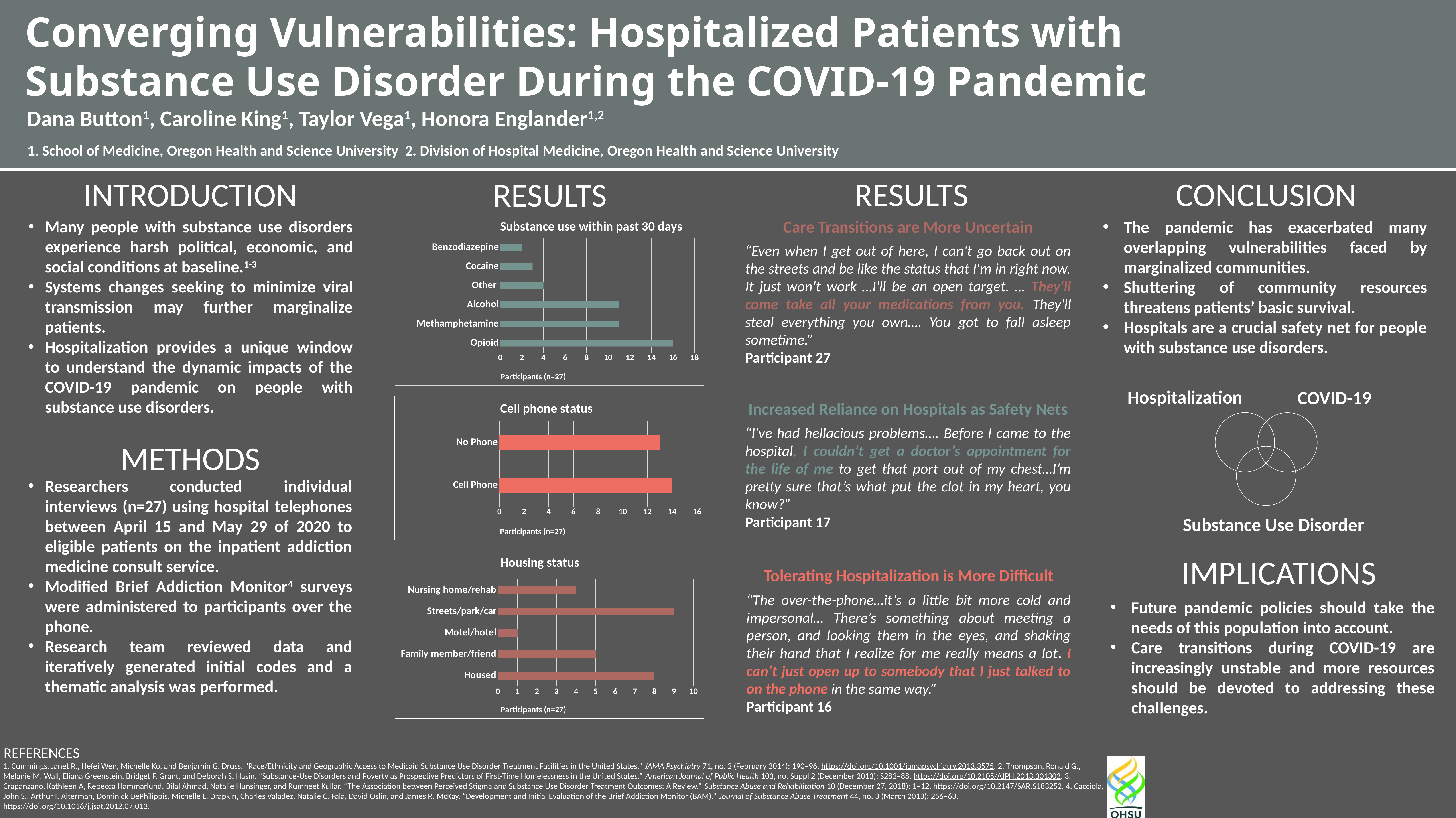

Converging Vulnerabilities: Hospitalized Patients with Substance Use Disorder During the COVID-19 Pandemic
Dana Button1, Caroline King1, Taylor Vega1, Honora Englander1,2
1. School of Medicine, Oregon Health and Science University 2. Division of Hospital Medicine, Oregon Health and Science University
CONCLUSION
RESULTS
INTRODUCTION
RESULTS
### Chart: Substance use within past 30 days
| Category | |
|---|---|
| Opioid | 16.0 |
| Methamphetamine | 11.0 |
| Alcohol | 11.0 |
| Other | 4.0 |
| Cocaine | 3.0 |
| Benzodiazepine | 2.0 |Many people with substance use disorders experience harsh political, economic, and social conditions at baseline.1-3
Systems changes seeking to minimize viral transmission may further marginalize patients.
Hospitalization provides a unique window to understand the dynamic impacts of the COVID-19 pandemic on people with substance use disorders.
Care Transitions are More Uncertain
The pandemic has exacerbated many overlapping vulnerabilities faced by marginalized communities.
Shuttering of community resources threatens patients’ basic survival.
Hospitals are a crucial safety net for people with substance use disorders.
“Even when I get out of here, I can't go back out on the streets and be like the status that I'm in right now. It just won't work …I'll be an open target. … They'll come take all your medications from you. They'll steal everything you own…. You got to fall asleep sometime.”
Participant 27
Hospitalization
COVID-19
### Chart: Cell phone status
| Category | |
|---|---|
| Cell Phone | 14.0 |
| No Phone | 13.0 |Increased Reliance on Hospitals as Safety Nets
“I've had hellacious problems…. Before I came to the hospital, I couldn’t get a doctor’s appointment for the life of me to get that port out of my chest…I’m pretty sure that’s what put the clot in my heart, you know?”
Participant 17
METHODS
Researchers conducted individual interviews (n=27) using hospital telephones between April 15 and May 29 of 2020 to eligible patients on the inpatient addiction medicine consult service.
Modified Brief Addiction Monitor4 surveys were administered to participants over the phone.
Research team reviewed data and iteratively generated initial codes and a thematic analysis was performed.
Substance Use Disorder
IMPLICATIONS
### Chart: Housing status
| Category | |
|---|---|
| Housed | 8.0 |
| Family member/friend | 5.0 |
| Motel/hotel | 1.0 |
| Streets/park/car | 9.0 |
| Nursing home/rehab | 4.0 |Tolerating Hospitalization is More Difficult
“The over-the-phone…it’s a little bit more cold and impersonal… There’s something about meeting a person, and looking them in the eyes, and shaking their hand that I realize for me really means a lot. I can’t just open up to somebody that I just talked to on the phone in the same way.”
Participant 16
Future pandemic policies should take the needs of this population into account.
Care transitions during COVID-19 are increasingly unstable and more resources should be devoted to addressing these challenges.
REFERENCES
1. Cummings, Janet R., Hefei Wen, Michelle Ko, and Benjamin G. Druss. “Race/Ethnicity and Geographic Access to Medicaid Substance Use Disorder Treatment Facilities in the United States.” JAMA Psychiatry 71, no. 2 (February 2014): 190–96. https://doi.org/10.1001/jamapsychiatry.2013.3575. 2. Thompson, Ronald G., Melanie M. Wall, Eliana Greenstein, Bridget F. Grant, and Deborah S. Hasin. “Substance-Use Disorders and Poverty as Prospective Predictors of First-Time Homelessness in the United States.” American Journal of Public Health 103, no. Suppl 2 (December 2013): S282–88. https://doi.org/10.2105/AJPH.2013.301302. 3. Crapanzano, Kathleen A, Rebecca Hammarlund, Bilal Ahmad, Natalie Hunsinger, and Rumneet Kullar. “The Association between Perceived Stigma and Substance Use Disorder Treatment Outcomes: A Review.” Substance Abuse and Rehabilitation 10 (December 27, 2018): 1–12. https://doi.org/10.2147/SAR.S183252. 4. Cacciola, John S., Arthur I. Alterman, Dominick DePhilippis, Michelle L. Drapkin, Charles Valadez, Natalie C. Fala, David Oslin, and James R. McKay. “Development and Initial Evaluation of the Brief Addiction Monitor (BAM).” Journal of Substance Abuse Treatment 44, no. 3 (March 2013): 256–63. https://doi.org/10.1016/j.jsat.2012.07.013.
